# Leaf extracts of eight selected southern African medicinal plants modulate pro-inflammatory cytokine secretion in LPS-stimulated RAW 264.7 macrophages

**DOI:** 10.1007/s10787-023-01420-9

**Published:** 2024-02-04

**Authors:** Gugulethu P. Khumalo, Wendy Loa-Kum-Cheung, Ben-Erik Van Wyk, Yunjiang Feng, Ian E. Cock

**Affiliations:** 1 Centre for Planetary Health and Food Security, Nathan Campus, Griffith University, 170 Kessels Rd, Nathan, QLD 4111 Australia; 2 School of Environment and Science, Nathan Campus, Griffith University, 170 Kessels Rd, Nathan, QLD 4111 Australia; 3 Department of Botany and Plant Biotechnology, University of Johannesburg, Auckland Park, P.O. Box 524, Johannesburg, 2006 South Africa; 4 Griffith Institute for Drug Discovery, Griffith University, 46 Don Young Rd, Nathan, QLD 4111 Australia

**Keywords:** Tumour necrosis factor, Interleukins, Traditional medicine, Ethnopharmacology, Anti-inflammatory activity

## Abstract

This study investigates the anti-inflammatory properties of extracts prepared from the leaves of eight southern African medicinal plants used traditionally to treat inflammation and pain. The inhibitory effect of aqueous and ethanol extracts on the release of pro-inflammatory cytokines was determined in lipopolysaccharide (LPS) stimulated and unstimulated RAW 264.7 murine macrophage cells. The levels of interleukin (IL)-1β, IL-6, tumour necrosis factor-α (TNF-α), interferon-gamma (IFN-γ), monocyte chemoattractant protein 1 (MCP-1) and macrophage inflammatory protein (MIP)-2 release were determined using cytokine multiplex-bead assays. The ethanol extracts of *Melianthus comosus* Vahl (commonly known as honey flower), *Tetradenia riparia* (Hochst.) Codd (misty plume bush) and *Warburgia salutaris* (G. Bertol.) Chiov. (pepper-bark tree), demonstrated the most significant inhibitory activity, with over 50-fold inhibition of IL-1β, IL-6 and TNF-α levels in LPS-stimulated RAW 264.7 macrophages. The aqueous extract of *M. comosus* also significantly inhibited the secretion of all the tested cytokines and chemokines. Phytochemical investigation of *M. comosus* ethanol leaf extract using ultra-high-performance liquid chromatography coupled with high-resolution mass spectrometry (UHPLC-HRMS) led to the detection of crassolide, deoxylimonoic acid D-ring-lactone, 2-hydroxynonanoic acid and 5-noniloxytryptamine. To the best of our knowledge, the cytokine inhibition properties of most of the medicinal plants screened in this study are reported for the first time. Our results support the use of southern African medicinal plants as anti-inflammatory remedies and provide an insight into the immunomodulatory mechanisms of action.

## Introduction

Inflammation is a beneficial defence mechanism against tissue damage. Unregulated activation of transcription factors and signalling molecules following tissue injury may progress into the development of chronic inflammation (Fullerton and Gilroy 2016). Cytokines are communication signals that regulate various biological processes, including cell function, proliferation, differentiation, and apoptosis (Dinarello 2011, 2018; Landskron et al. 2014). The cytokines interleukin (IL)-1β, IL-6 and TNF-α are classified as principal pro-inflammatory mediators that have been identified as key therapeutic targets in resolving inflammation (Hirano 2021; Laczko et al. 2020; Scheller et al. 2011). Whilst there is a global increase in the use of natural products as therapeutics, a significant decline in indigenous knowledge of medicinal plant use remains a major concern in southern Africa (Van Wyk and Gericke 2018). Modern lifestyles have directed most of the younger generation away from traditional therapies and towards allopathic medicine (Ndhlala et al. 2011; Magwede et al. 2019).

In southern African, medicinal plant use to treat a variety of chronic inflammatory diseases is a common practice. Medicinal plant products are popularly sold as raw plant material at informal (muthi) markets, with most traders being rural inhabitants who depend on the informal trade of plant material to financially sustain their families (Williams et al. 2004; Mander et al. 2007; Khumalo 2018). These medicines are commonly prepared with water and taken as decoctions and infusions, or they may be applied externally as raw plant material in powdered or poultice forms to treat various inflammatory conditions (Khumalo et al. 2022). However, many of these medicines lack biological and toxicity studies to account for their traditional use as analgesic and anti-inflammatory remedies (Elgorashi and McGaw 2019; Khumalo et al. 2022). This study evaluates the cytokine inhibition properties of leaf extracts prepared from eight southern African medicinal plants that are commonly used traditionally to treat pain and inflammation (Khumalo et al. 2022). The cytokine multiplex-bead assay was used to determine the immune-modulatory properties of plant extracts against the pro-inflammatory cytokines IL-1β, IL-6, TNF-α, inteferon (IFN)-γ, as well as the chemokines monocyte chemoattractant protein-1 (MCP-1) and macrophage inflammatory protein-2 (MIP-2) in lipopolysaccharide (LPS)-induced RAW 264.7 murine macrophages.

The eight medicinal plant species screened in this study were chosen from a list of 555 species that were identified in a recent review to be used traditionally against inflammatory and pain-related conditions in southern Africa (Khumalo et al. 2022). The recorded inflammatory conditions included general inflammation, pain, toothache, headache, rheumatism, oedema or swellings, general body pains, earache, backache, arthritis, abdominal pains, chest pains, internal body pains, haemorrhoids, labour pains and rheumatic fever (Khumalo et al. 2022). These species were selected based on their frequent use and popularity across the 22 main ethnic groups of southern Africa. Furthermore, whilst the selected medicinal plants are widely commercialised, the ethnopharmacological studies investigating the possible mechanism of action as immunoregulators are limited. Therefore, the pro-inflammatory cytokine inhibition properties of most species are reported for the first time in this study.

## Materials and methods

### Plant collection and extraction

Fresh leaves of *Harpagophytum procumbens *(Burch.) DC ex Meisn. (commonly called Devil’s claw; voucher code JRAU_HP), *Melianthus comosus* Vahl. (honey flower; voucher code JRAU_MC) *Mentha longifolia* (L.) Huds. (horse mint; voucher code JRAU_ML), *Plumbago auriculata* Lam. (Cape Plumbago; voucher code JRAU_CP), *Terminalia sericea* Burch. ex DC. (silver cluster-leaf; voucher code JRAU_TS), *Tetradenia riparia* (Hochst.) Codd, (misty plume bush; voucher code JRAU_TR) *Warburgia salutaris* (G. Bertol.) Chiov. (pepper-bark tree; voucher code JRAU_WS), and *Zantedeschia albomaculata* (Hook.) Baill. (Calla lily; voucher code JRAU_ZA)*,* were collected from fully grown feature specimens planted on the University of Johannesburg, Auckland Park campus in April 2021. The leaves were allowed to air dry at room temperature in a greenhouse in the shade for 2 weeks. Voucher specimens for each of the plant species were prepared and deposited at the University of Johannesburg herbarium. The plant parts tested (leaves) were chosen based on their traditional use to treat pain and inflammatory conditions and were verified by Prof B.-E van Wyk of the University of Johannesburg.

### Preparation of plant extracts

Distilled water and ethanol were used to prepare extracts of the eight plant species. All solvents were obtained from Ajax Fine Chemicals, Australia and were AR grade. Dried and powdered leaf material (5 g) was soaked individually in 50 mL (1:50 w/v) of each solvent for 24 h at room temperature. Extraction with distilled water was chosen to mimic the method that medicinal plants are prepared traditionally as infusions and decoctions. Ethanol was selected as a solvent to obtain a significant quantity of non-polar (as well as polar) constituents that may otherwise be absent in aqueous extracts. All extracts were subsequently filtered using Whatman No. 1 filter paper and allowed to evaporate to dryness in a fume hood.

Dried extracts were resuspended in 10 mL deionised water containing 1% dimethylsulfoxide (DMSO). Powdered *Curcumin longa* L. (commonly known as turmeric; Super Strength Bio Turmeric 3100) was purchased from Healthy Care, Australia (Batch no. 750710) as individual capsules. Each capsule contained 155 mg of turmeric dried plant material, which is standardised to contain the equivalent to 100 mg curcumin. The material from one capsule was suspended in 4 mL DMSO and then 28 mL distilled water was added to prepare a stock solution of 5 mg/mL (12.5% DMSO). Turmeric extract was used at a concentration of 1.25 cmg/mL (containing 3.12% DMSO) as a positive control for the cytokine assays. Curcumin is a major component of turmeric derived from *Curcuma longa *L., (Fürst and Zündorf 2014). The mechanism of action in relation to its anti-inflammatory properties has been widely explored. Curcumin is a potent inhibitor of pro-inflammatory signalling pathways, including the NFκB, MAPK, COX, and LOX pathways, and downregulates the secretion of cytokines, and inhibits the expression of cell adhesion molecules (Fürst and Zündorf 2014).

### Cell culture and treatment

RAW 264.7 murine mouse macrophages (TIB-71) were selected as a model system as they are widely used to study cytokine modulation, whilst human dermal fibroblasts (PCS-201-010) were used for cell viability assays. Both cell lines were purchased from the American Type Culture Collection (ATCC). Both cell lines were cultured in a high-glucose Dulbecco’s Modified Eagle’s medium (DMED, Gibco) supplemented with 100 µg/mL penicillin/streptomycin (Gibco) and 10% fetal bovine serum (FBS, Gibco). Cell cultures were maintained in a 5% CO_2_ incubator with 70% humidity at 37 °C until 80% confluence was achieved. LPS was purchased from Sigma-Aldrich and used to stimulate an inflammatory response at a concentration of 100 ng/mL. The cells were passaged twice a week and the experiments were conducted following the fourth passage.

### Measurement of cell viability by MTS assay

The MTS assay (3-(4,5-dimethylthiazol-2-yl)-5-(3-carboxymethoxyphenyl)-2-(4-sulfophenyl)-2H-tetrazolium) was performed as previously described by Riss et al. (2016) to determine the appropriate extract dose for biological screening. Briefly, a 100 µL volume of cells (1 × 10^6^ cells/well) were seeded in each well of a sterile 96-well micro-titre plate and incubated at 37 °C for 24 h in a humidified environment with 5% CO_2_. All samples were screened in duplicate. Cells without exposure to plant extracts served as negative controls, whilst cells treated with 10% DMSO were used as a positive control. After 24 h, the cells were exposed to 100 µL volumes of varying concentrations (5, 2.5, 1.25, 0.62 and 0.31 mg/mL) of the plant extracts and incubated at 37 °C for a further 24 h. Following 48 h incubation, the old media containing the extracts was discarded, and 100 µL of fresh media was added, followed by 25 µL of CellTiter 96^®^ Aqueous One Solution Reagent (Promega, Australia). After another 1.5-h incubation, the colour change of the MTS solution from yellowish green to dark red/brown was measured at 490 nm using a Molecular Devices Spectra Max M3 plate reader.

### Cytokine multiplex-bead assay

The effects of the extracts on cytokine secretion were assessed under two conditions. Firstly, cytokine levels in unstimulated RAW 264.7 cells were used as a negative control to determine the basal cytokine release upon exposure to the extracts. The effects of the extracts and controls on cellular immunity were also examined by activating RAW 264.7 with 100 ng/mL LPS (Sigma-Aldrich). For all tests, media containing approximately 1 × 10^6^ cells in 3 mL of DMED media were seeded into the wells of flat-bottom 6-well culture plates. After 24 h incubation, the cells were treated with 2.5 mg/mL or 1.25 mg/mL of the plant extracts 3 h prior to the addition of LPS. The choice of extract concentration was influenced by the MTS assay results of the plant extracts. Aqueous and ethanol extracts that exhibited > 80% cell viability at 2.5 mg/mL were screened at that concentration for their immunomodulatory effects. Some ethanol extracts induced substantial decreases in cell viability at 2.5 mg/mL and were, therefore, screened at 1.25 mg/mL. The LPS-stimulated RAW 264.7 cells were also treated in duplicate with 1.25 mg/mL turmeric as a positive control. Other untreated cells were included on each plate as negative controls. The plates were incubated for 48 h at 37 °C in a 5% CO_2_ incubator with 70% humidity. Following the 48 h incubation, the culture supernatants were collected for cytokine analysis.

All multiplex-bead assays were performed in duplicate using customised Multiplex Bead Assays (ThermoFisher, Australia), which are designed to detect multiple murine-specific proteins simultaneously. The levels of IL-1β, IL-6, TNF-α, INF-γ, MCP-1 and MIP-2 were quantified according to the manufacturers’ instructions and performed at Cardinal Bioresearch, Australia on a Luminex 200 flow-cytometry system (Milliplex^®^, Australia). Cytokine concentrations (in pg/mL) were calculated based on the sample fluorescence intensity (MFI), compared to cytokine standard curves and are expressed as a % of the untreated RAW cells. All tests were performed in duplicate and are expressed as mean ± standard error of the mean (SEM).

### Chemical studies

The ethanol leaf extract of *M. comosus* was further studied for the presence of possible bioactive constituents using UHPLC-HRMS analyses based on its potent inhibition of secretion of all of the tested cytokines and chemokines screened in this study.

### UHPLC-HRMS conditions

The ethanol extract was subjected to UHPLC-HRMS analysis (5 μL injection volume). UHPLC-HRMS run were performed with an Ultimate 3000 RS UHPLC coupled to a Bruker maXis II ETD ESI-qTOF mass spectrometer using an analytical Thermo Scientific Accucore C18 column (150 × 2.1 mm, 2.6 mm, 80 Å). An isocratic elution using 10% methanol (0.1% formic acid) in H_2_O (0.1% formic acid) was held for 1 min, followed by a linear gradient of 10% methanol (0.1% formic acid) in H_2_O (0.1% formic acid) to 100% methanol (0.1% formic acid) over 9 min. This column was then washed with 100% methanol (0.1% formic acid) for 2.5 min before switching back to 10% methanol (0.1% formic acid) in H_2_O (0.1% formic acid) over 0.2 min and re-equilibrating the column for 3 min. This provided an LC run of 15 min, conducted at a flow rate of 0.3 mL/min.

### UHPLC-HRMS data analyses

The generated UHPLC-MS data were analysed using DataAnalysis 5.2 (Bruker Daltonics) and dereplicated using Compound Crawler 3.1 (Bruker Daltonics) with access to Chemical Entities of Biological Interest (ChEBI) database to putatively identify the presence of some compounds in the plant extract based on *m/z*, mass spectral properties and retention index. The UV chromatogram was recorded at the wavelength of 254 nm.

## Results

### MTS assays

**Fig. 1:**
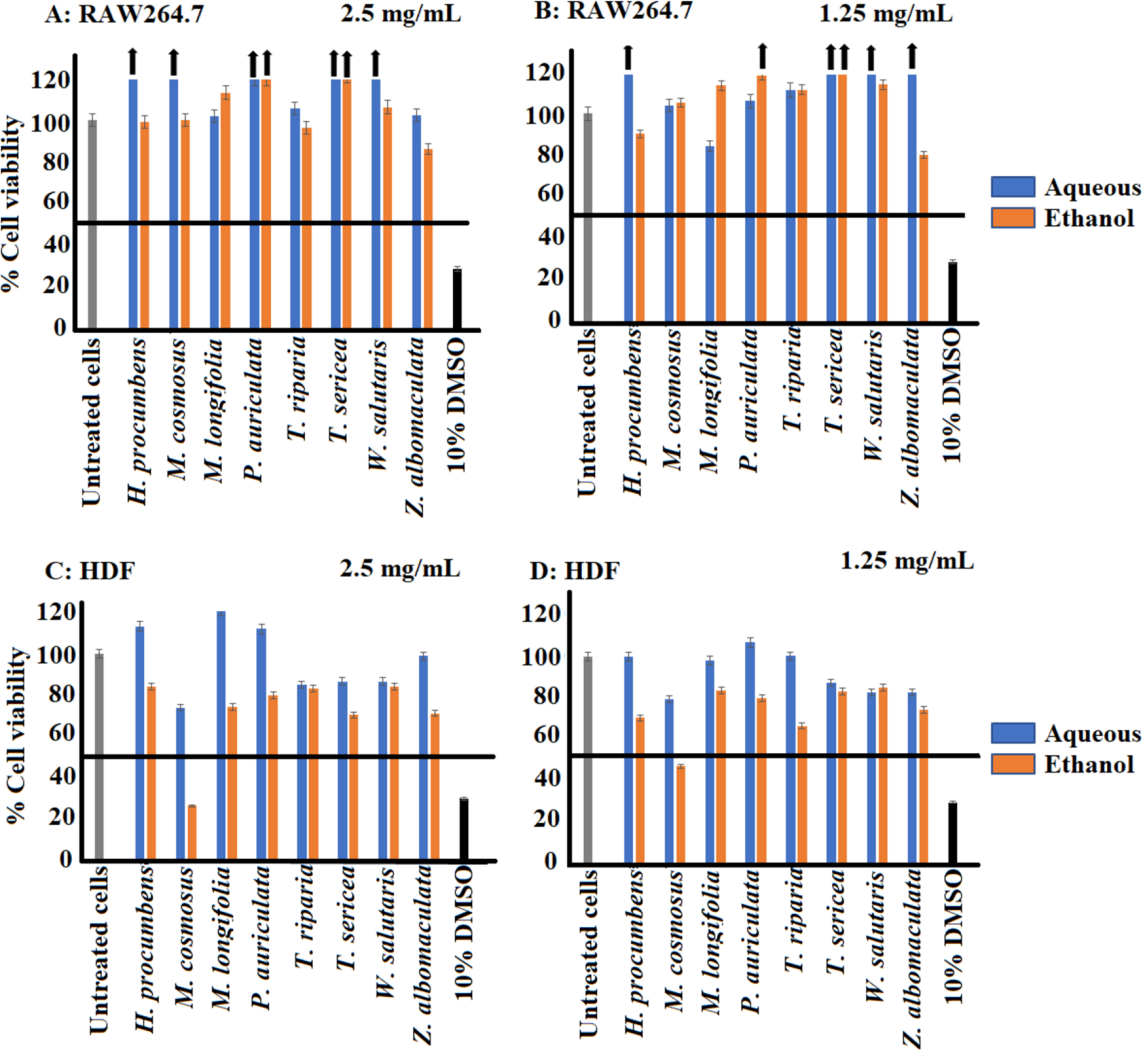
The cytotoxic effects of the selected southern African medicinal plants in RAW 264.7 macrophages and human dermal fibroblast at 2.5 and 1.25 mg/mL was determined using the MTS assay. **A**,** B** Aqueous and ethanol extracts tested in RAW 264.7 macrophage cells; **C**, **D** Aqueous and ethanol extracts of in human dermal fibroblast (HDF) cells. (mean ± SEM of duplicates). The arrows indicate percentage cell viabilities of plant extracts > 120% and the horizontal lines indicate 50% viability

The MTS assay was used to determine the percentage cell viability of aqueous and ethanol extracts at 2.5 mg/mL and 1.25 mg/mL against RAW 264.7 murine macrophages (Fig. 1A and B) and human dermal fibroblasts (HDF; Fig. 1C and D). The MTS assay was performed using varying extract concentrations. At 2.5 mg/mL, all the aqueous extracts exhibited ≥ 80% cell viability against both tested cell lines, therefore this was the concentration used to perform the cytokine assays of all the aqueous plant extracts. The ethanol extracts that displayed potential toxicities (< 50% cell viability) at 2.5 mg/mL against macrophages and fibroblasts were tested at 1.25 mg/mL in the cytokine assay. Results obtained from this study showed that all the aqueous and ethanol extracts were non-toxic at 1.25 mg/ml against RAW 264.7 and HDF cells, with the exception of *M. comosus*. The ethanol extract of *M. comosus* exhibited toxicity against human dermal fibroblasts with < 50% cell viability at both concentrations tested (Fig. 1C and D). However, RAW 264.7 macrophages treated with the ethanol and aqueous extracts at 2.5 mg/mL and 1.25 mg/mL exhibited no toxicities in the MTS assay (Fig. 1A and B). In addition, medicinal plant extracts of *H. procumbens*, *T. sericea*, *W. salutaris* and *Z. albomaculata* exhibited proliferative effects, with the greatest percentage cell viability of 164% observed from the aqueous extract of *H. procumbens* against RAW 264.7 macrophages.

### Cytokine assays in RAW 264.7 cells in the absence of stimuli (LPS)

Cytokine levels (IL-1β, IL-6, TNF-α, INF-γ, MCP-1 and MIP-2) were determined in RAW 264.7 cells without LPS stimulation upon exposure to the extracts. This was undertaken to investigate the immunomodulatory properties of medicinal plant extracts in the absence of an inflammatory stimulus. The levels of cytokine expression in untreated cells (control) were very low and, therefore, no significant cytokine inhibition was evident upon extract exposure. Therefore, these results are not presented herein. Instead, the results presented in this study show the anti-inflammatory properties of plant extracts in LPS-stimulated RAW 264.7 murine macrophages.

### Cytokine assays in LPS-stimulated RAW 264.7 macrophages

#### Interleukin-1 (IL-β) inhibition activity

**Fig. 2:**
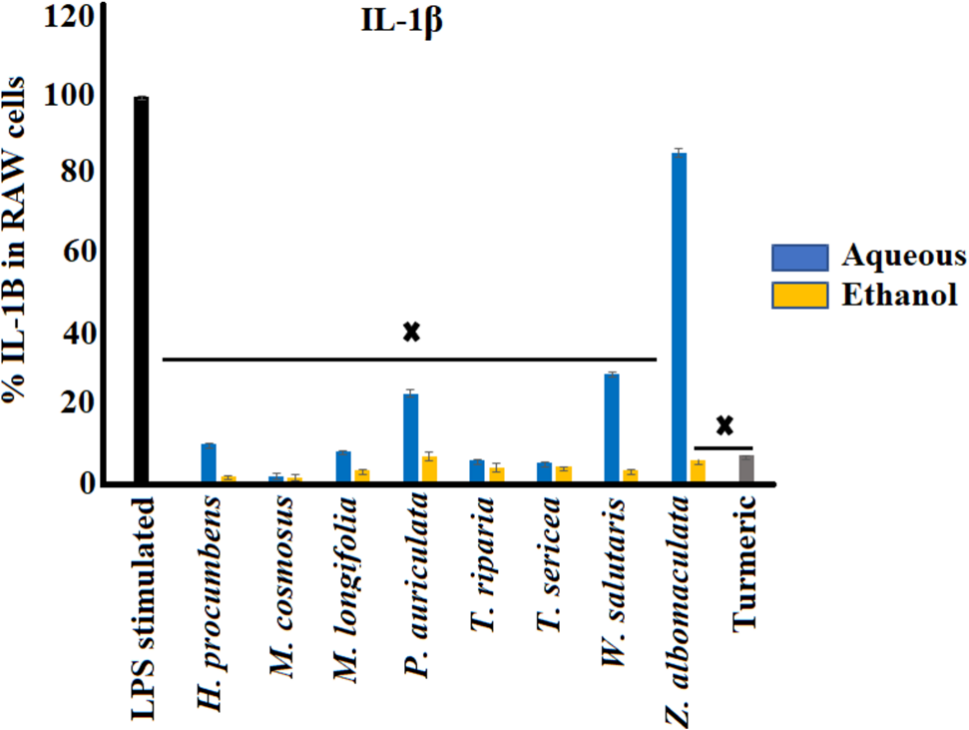
Effect of selected southern African medicinal plants on interleukin-1β secretion in LPS-stimulated (100 ng/mL) RAW 264.7 cells (mean ± SEM of duplicates). X indicates statistical inhibition differences to the LPS control (*p* < 0.005)

The leaf extracts of selected medicinal plants were evaluated for their IL-1β activity in LPS-stimulated RAW 264.7 cells (Fig. 2). The RAW 264.7 murine macrophage cells were treated with aqueous and ethanol extracts of eight medicinal plants 3 h prior to treatment with LPS (100 ng/mL). Cells treated with LPS in the absence of extracts (negative control) showed increased levels of IL-1β production. All the plant extracts showed decreases (*p* < 0.005) in IL-1β levels compared to the control, with the exception of the aqueous extract of *Z. albomaculata,* which only showed an apparent decrease. Furthermore, all the ethanol extracts exhibited significant inhibition (*p* < 0.005) of IL-1β, with over 90% decreases compared to the control.

The potent IL-1β inhibition of medicinal plant extracts from cells treated with the aqueous extracts of *H. procumbens*, *M. comosus*, *M. longifolia*, *T. riparia* and *T. sericea* may justify the traditional method of preparation using water as infusions and decoctions. *Melianthus comosus* displayed the greatest anti-inflammatory activity against IL-1β, with inhibition by 97.9% and 98.1% from the aqueous and ethanol extracts, respectively. Furthermore, most of the ethanol extracts substantially decreased IL-1β levels compared to the positive control.

#### Inteleukin-6 (IL-6) inhibition activity

**Fig. 3:**
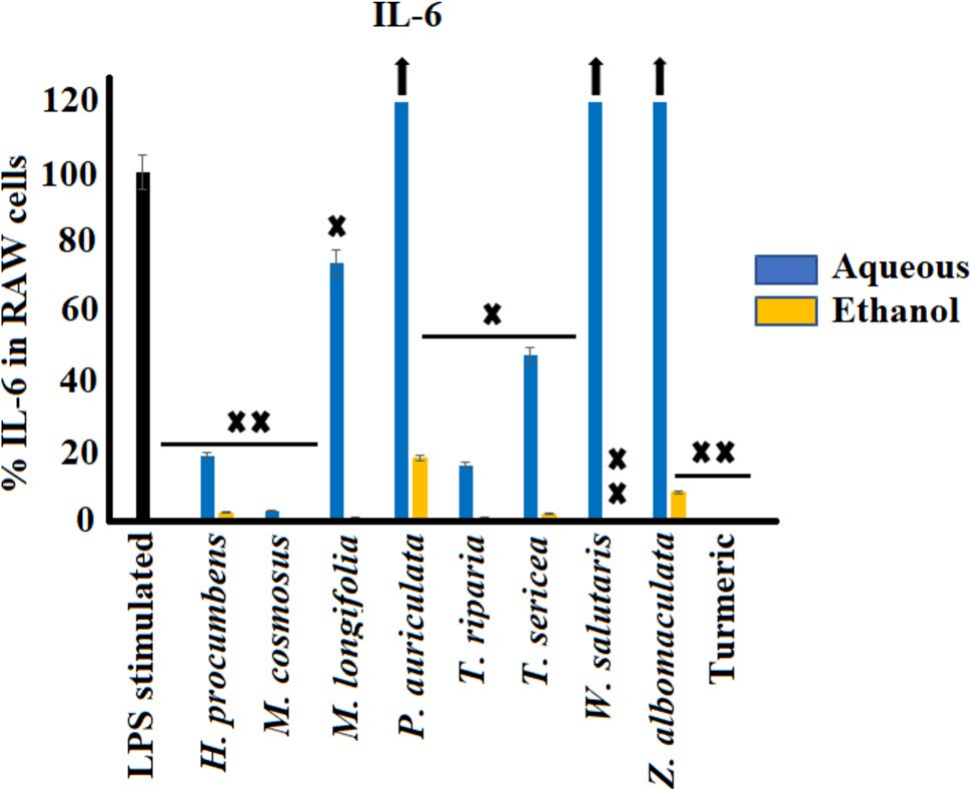
Cytokine inhibitory effects of the selected southern African medicinal plants against Interleukin-6 secretion in LPS-stimulated RAW 264.7 murine macrophage cell line (mean ± SEM of duplicates). Black arrows indicate percentage inhibition of plant extracts is greater than the axis scale of the graphs. X (*p* < 0.005); and XX (*p* < 0.0005) indicate statistical inhibition difference from the LPS control, respectively

The effect of the leaf extracts of eight medicinal plants on interleukin-6 inhibition were determined in LPS-induced RAW 264.7 cells (Fig. 3). LPS-stimulated cells in the absence of plant extracts (negative control) showed substantially increased IL-6 production to approximately 2111.6 pg/mL (100%). In contrast to the aqueous extracts, all the ethanol extracts exhibited noteworthy IL-6 inhibition (*p* < 0.0005), with the greatest decrease noted for the *M. comosus* extracts (99.7%), followed by *W. salutaris* (99.4%) and *T. riparia* (98.9%) compared to the control. Notably, the aqueous leaf extracts of *P. auriculata*, *W. salutaris* and *Z. albomaculata* exhibited substantial pro-inflammatory effects by significantly enhancing the release of IL-6, with the highest increase from *Z. albomaculata* up to 16 791.4 pg/mL (795.2%) compared to the control.

The aqueous and ethanol extracts of *H. procumbens, M. comosus* and *T. riparia* significantly inhibited IL-6 secretion in LPS-stimulated RAW 264.7 cells. This indicates that the bioactivity of these medicinal plants is largely influenced by the polar constituents. Furthermore, the IL-6 inhibition activity of *M. comosus* was more active than the positive turmeric control, significantly decreasing IL-6 levels from 2111.6 pg/mL in the negative control to 6.5 pg/mL in cells treated with the ethanol extract, indicating a > 300-fold decrease in IL-6 secretion.

#### Tumour necrosis factor-α (TNF-α) inhibition activity

**Fig. 4:**
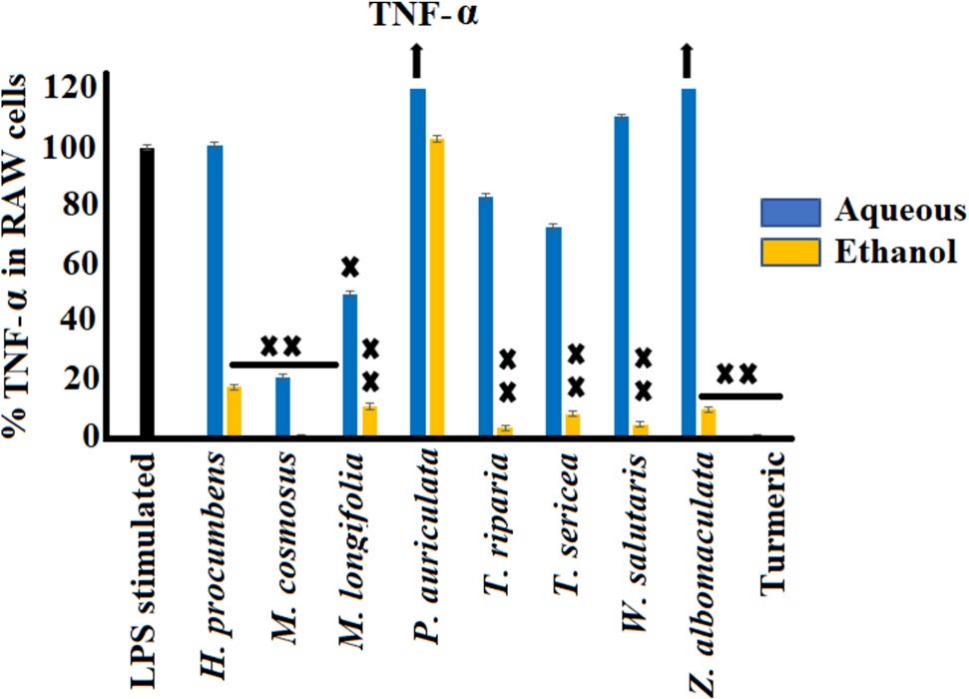
The anti-inflammatory effects of selected southern African medicinal plants against TNF-α in LPS-stimulated (100 ng/mL) RAW 264.7 murine macrophage cell line (mean ± SEM of duplicates). Black arrows indicate percentage inhibition of plant extracts is greater than the axis scale of the graphs. X (*p* < 0.005); and XX (*p* < 0.0005) indicate statistical inhibition difference from the LPS control, respectively

The selected southern African medicinal plants were evaluated for their anti-inflammatory properties against tumour necrosis factor-α in LPS-induced RAW 264.7 cells (Fig. 4). The LPS-stimulated cells (100 ng/mL) in the absence of extracts (negative control) substantially enhanced the production of TNF-α levels up to 3027.9 pg/mL (100%). All the ethanol extracts showed significant inhibition (*p* < 0.0005) of TNF-α levels, in contrast to the aqueous extracts, with the exception of the *P. auriculata* extract*.* Whilst the aqueous extract of *P. auriculata* displayed the greatest up-regulation of TNF-α levels to 158.5% compared to the untreated control (*p* < 0.005), the ethanol extract showed no significant effect on TNF-α secretion compared to the control.

In addition, the aqueous extracts of *Z. albomaculata* and *W. salutaris* induced increases in TNF-α levels up to 133.8% and 110.5%, respectively, compared to the control. *Melianthus comosus* showed the greatest inhibition of TNF-α levels (*p* < 0.005), from 3027.9 pg/mL in the control to 0.18 pg/mL (approximately 16 000-fold decrease) in cells treated with the ethanol leaf extract, corresponding to a 99.8% inhibition of TNF-α in LPS-stimulated RAW 264.7 cells (*p* < 0.005). Furthermore, the ethanol extracts of *T. riparia* and *W. salutaris* displayed significant TNF-α inhibition of up to > 95% compared to the control (*p* < 0.005). Whilst most aqueous extracts exhibited no apparent inhibition or further enhanced TNF-α secretion, the aqueous extracts of *M. comosus* and *M. longifolia* displayed substantial inhibition by a fivefold and twofold decrease in TNF-α levels, respectively, compared to the control (*p* < 0.005).

#### Interferon-gamma (IFN-γ) inhibition activity

**Fig. 5:**
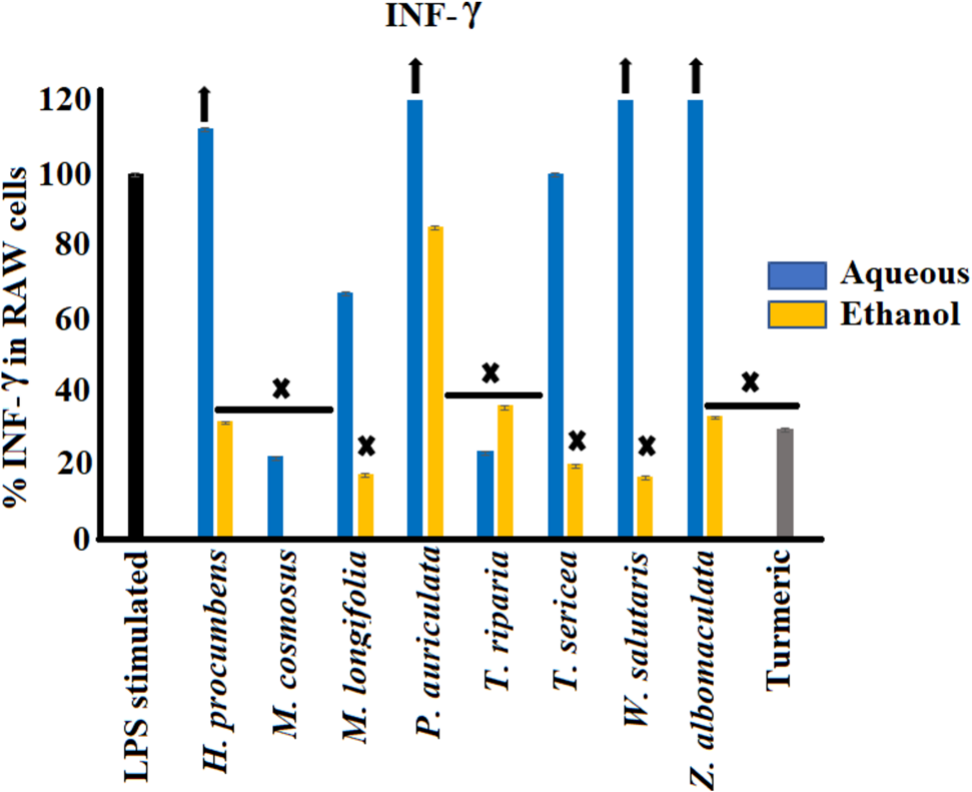
Interferon-gamma inhibition activity of plant extracts of the selected southern African medicinal plants in LPS-stimulated (100 ng/mL) RAW 264.7 murine macrophage cell line (mean ± SEM of duplicates). Black arrows indicate percentage inhibition of plant extracts is greater than the axis scale of the graphs. X (*p* < 0.005) indicates statistical inhibition difference from the LPS control

Aqueous and ethanol leaf extracts of the tested plant species of southern Africa were screened for their anti-inflammatory properties against IFN-γ in LPS-stimulated RAW 264.7 macrophage cells (Fig. 5). The ethanol plant extracts showed significant inhibition of LPS-induced IFN-γ levels compared to the aqueous extracts, with the exception of *P. auriculata*, which showed an apparent decrease (although this was not statistically significant). The greatest IFN-γ inhibition activity was observed from the ethanol extract of *M. comosus* with 100% inhibition, while the aqueous extract also showed a substantial decrease of up to 77.8% IFN-γ secretion compared to the control (*p* < 0.005). Furthermore, cells treated with the aqueous extracts of *T. riparia* showed a significant decrease (*p* < 0.005) of IFN-γ levels compared to the control.

The aqueous extracts of most medicinal plants including *H. procumbens*, *P. auriculata*, *W. salutaris* and *Z. albomaculata* exhibited an increase of over 100% of IFN-γ levels in LPS-induced RAW 264.7 cells compared to the control. The strongest increase was noted from the aqueous extracts of *Z. albomaculata* and *W. salutaris*, with up to 365.3% and 182.6% of the control value, respectively.

### Chemokine activity of aqueous and ethanol extracts against MCP-1 and MIP-2 secretion

**Fig. 6:**
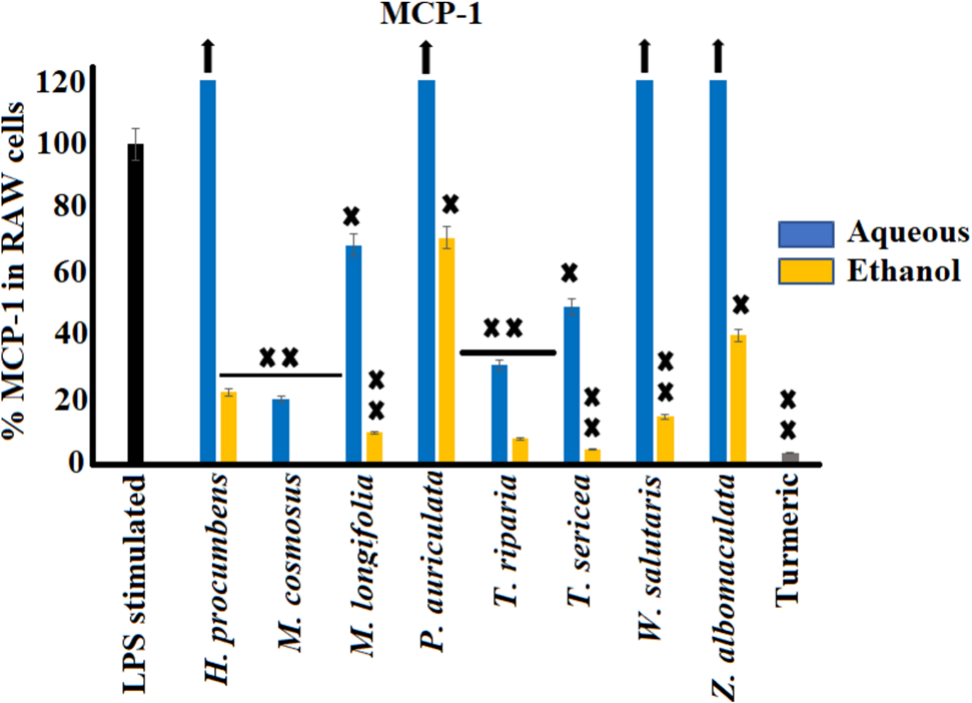
Effects of eight selected medicinal plants against MCP-1 activity in LPS-stimulated (100 ng/mL) RAW 264.7 murine macrophage cell line (mean ± SEM of duplicates). Black arrows indicate percentage inhibition of plant extracts is greater than the axis scale of the graphs. X (*p* < 0.005); and XX (*p* < 0.0005) indicate statistical inhibition difference from the LPS control, respectively

**Fig. 7:**
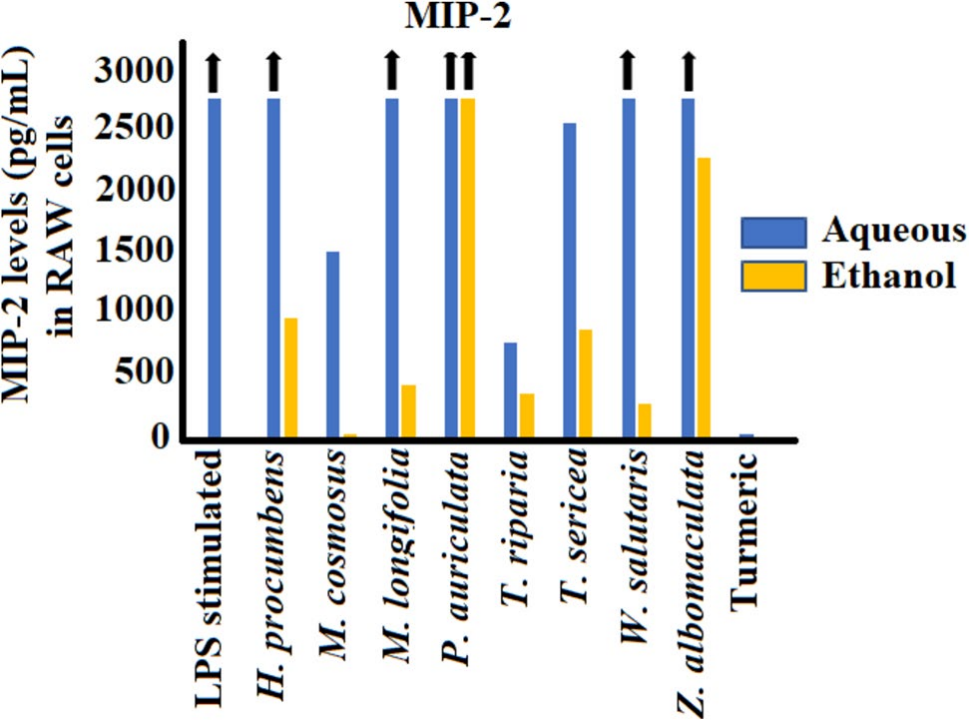
Chemokine inhibition activity (pg/mL) of leaf extracts of the selected southern African medicinal plants against MIP-2 in LPS-stimulated (100 ng/mL) RAW 264.7 murine macrophage cell line. Black arrows indicate percentage inhibition of plant extracts is greater than the axis scale of the graphs. Statistical differences of MIP-2 inhibition of plant extracts to the LPS control could not be determined due to off-scale value of MIP-2 levels in the LPS-stimulated but untreated control

The selected medicinal plants traditionally used to treat pain and inflammation were also screened for modulation of the secretion of the chemokines MCP-1 and MIP-2 in LPS-induced RAW 264.7 macrophages (Figs. 6 and 7, respectively). The ethanol extracts showed substantially greater inhibition (*p* < 0.005) of MCP-1 compared to the aqueous extracts. The greatest MCP-1 inhibition was observed for the ethanol extracts of *M. comosus* and* T. sericea,* with 99.4% and 95.5% inhibition, respectively, compared to the control. The aqueous extracts from *H. procumbens*, *P. auriculata*, and *W. salutaris* favoured the up-regulation of MCP-1 release in LPS-stimulated raw 264.7 cells. The greatest increase was observed from the aqueous extract of *P. auriculata,* with up to 572.7% of MCP-1 release compared to the control.

The MIP-2 percentage data could not be calculated since the LPS stimulation of RAW 264.7 cells increased the MIP-2 levels to > 3000 pg/mL, which was beyond detectable range of the assay. Therefore, only the mean averages of raw data are presented herein as pg/mL in Fig. 7. In contrast to the aqueous extracts, the ethanol extracts exhibited substantial inhibition activity of MIP-2 levels compared to the control, with the exception of *P. auriculata*, which had high MIP-2 levels > 3000 pg/mL. RAW 264.7 cells treated with the ethanol extract of *M. comosus* showed the greatest inhibition of MIP-2 levels from > 3000 pg/mL down to 14.96 pg/mL (*p* < 0.005).

### UHPLC-HRMS analyses of *M. cosmosus* extract

**Fig. 8:**
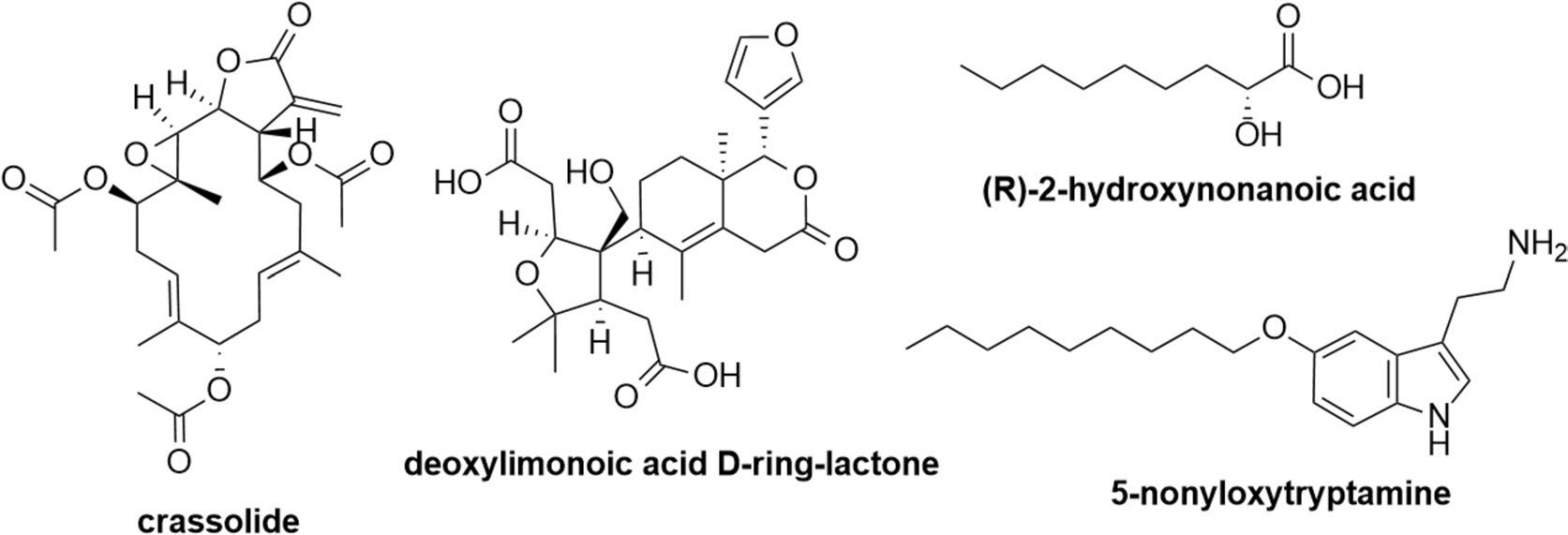
The structures of compounds putatively identified in the ethanolic leaf extract of *M. comosus*

Due to its strong inhibition of all of the pro-inflammatory cytokines and chemokines screened in this study, the *M. cosmosus* ethanol extract was selected for phytochemical analysis. Briefly, the UHPLC-HRMS data were acquired with an Ultimate 3000 RS UHPLC coupled to a Bruker maXis II ETD ESI-qTOF and the mass spectrum was calibrated externally with 0.1 mg/mL of sodium trifluoroacetate. Table 1 and Fig. 8 show the metabolites putatively identified in the ethanol leaf extract of *M. comosus*. The findings are limited to search engines including PubMed and SciFinder, and were dereplicated using Compound Crawler 3.1 (Bruker Daltonics) with access to Chemical Entities of Biological Interest (ChEBI) database).

**Table 1:** UHPLC-MS data of compounds putatively identified in the ethanolic *Melianthus cosmosus* leaf extract. The structures of the compounds are shown beneath the table

Peak	Retention time (*t*_R_, min)	[M+H^+^] *m/z*	[M+Na^+^] *m/z*	Molecular formula	No. of hits on ChEBI^a^	Putative identification^b^
A	9.05	491.2276		C_26_ H_34_O_9_	5	Crassolide and deoxylimonoic acid D-ring-lactone
B	9.50		197.1148	C_9_ H_18_O_3_	6	(R)-2-Hydroxynonanoic acid
C	10.65		325.2273	C_19_H_30_N_2_O	1	5-Noniloxytryptamine

^a^Accessed on June 2023

^b^Compounds with reported anti-inflammatory activity, identified as hits on CheBI

**Fig. 9:**
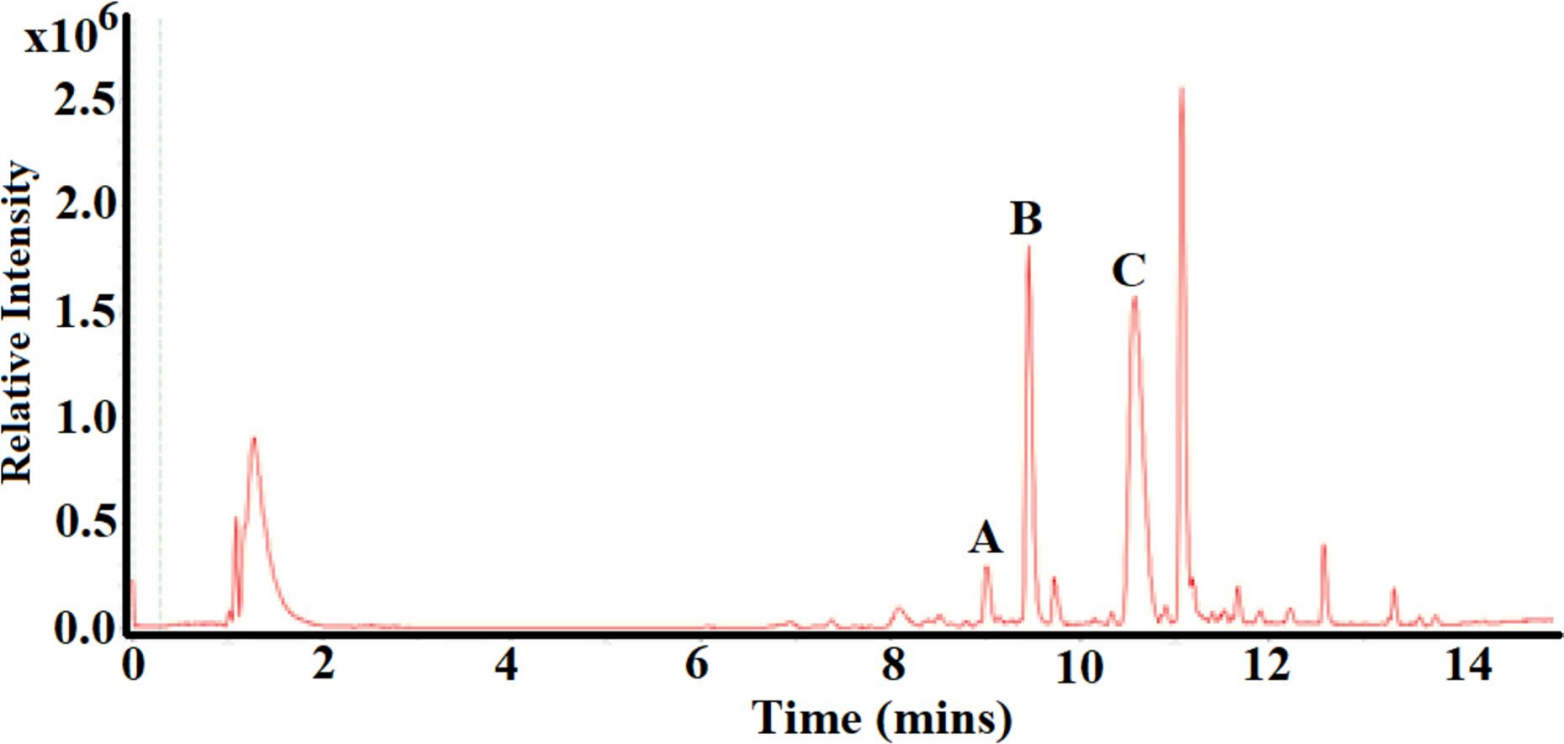
Base peak chromatogram (all MS) for the ethanol extract of *M. cosmosus*

Crassolide (peak A; Fig. 9) was putatively identified in the ethanol extract of *M. cosmosus* leaves. The diterpenoid has been reported by various authors to exhibit immunoregulatory properties (Chao et al. 2008; Lin et al. 2021; Lai et al. 2022). Crassolide has potent in vivo anti-inflammatory effects against an auto-immune disease known as antiphospholipid syndrome through significant inhibition of pro-inflammatory cytokines (including (TNF)-α, interleukin (IL)-6, IL-12 and IL-23) and dendritic cell maturation (Lin et al. 2021). Crassolide also exhibits antitumour effects against human H460 non-small-cell lung cancer cells through stimulation of reactive oxygen species (ROS) production, thereby promoting apoptosis (Lai et al. 2022).

2-Hydroxynonanoic acid (197.1172) (peak B; Fig. 9) modulates inflammation induced by microbial infections associated with *Pseudomonas* sp. and *Escherichia coli* (Pohanka et al. 2005; Van Der Hooft et al. 2019). *Pseudomonas* sp. contribute largely to respiratory tract infections and play a major role in the initiation and progression of inflammation through activation of innate immune signalling pathways by binding to TLR4-MD-2 receptors (Pier 2007; Qin et al. 2022).

Similarly, 5-noniloxytryptamine (peak C; Fig. 9) is a potent neuroprotective agent (Saini et al. 2016; Kalotra et al. 2020; Kalotra and Kaur 2021). Notably, 5-noniloxytryptamine mimics polysialic acid (PSA), which is required for inducing and maintaining plasticity and repair of the nervous system upon tissue injury (Doğanyiğit and Üner 2021). Notably, the potential presence of bioactive constituents reported from the ethanol extracts of *M. comosus* have not been previously identified for this species and therefore reported for the first time in this study. Previous studies have identified few *M. cosmosus* compounds, including the toxic bufadienolides (Van Wyk et al. 2009; Maroyi 2019; Bedane et al. 2020).

## Discussion

The anti-inflammatory effects of the leaves of eight medicinal plants were evaluated for the ability to modulate the secretion of several cytokines and chemokines in LPS-induced RAW 264.7 and unstimulated murine macrophage cells. Several of the plant extracts exhibited strong inhibition of pro-inflammatory cytokine and chemokines, thus supporting their traditional use as anti-inflammatory remedies. IL-1β is a pro-inflammatory cytokine that that induces pain, inflammation, and auto-immune disorders (Dinarello 2018). Two types of IL-1 (IL-1α and 1β) share the same receptor (IL-1R1) and require the presence of an additional protein (IL1 receptor accessory protein) (IL-1RAcP) to promote intracellular signalling during an inflammatory response (Dinarello 2018). Interleukin-1β injection in mice directly induces inflammatory pain and thermal hyperalgesia by enhancing gene expression of cyclooxygenase-2 (COX-2), and therefore the subsequent synthesis of prostaglandin E_2_ (PGE_2_) (Ji et al. 2018). Several monoclonal antibodies, including anakinra and canakinumab that target interleukin-1 activity through blocking IL-1 receptor have been clinically approved as monotherapies. Additionally, administering these antibodies increases recovery rates in patients suffering from rheumatologic conditions and heart failure (Fitzgerald et al. 2005; Dinarello 2011, 2018). However, due to the expensive cost of these therapies, they remain inaccessible to much of the population (Liu 2014). In contrast, medicinal plant products are cost effective, generally easily accessible and have been used since ancient times for various inflammatory disorders, often without substantial toxicities.

All the tested medicinal plants displayed potent anti-inflammatory activity against IL-1β secretion in RAW 264.7 cells. The aqueous and ethanol extracts of *Mentha longifolia* were particularly strong inhibitors of IL-1β secretion. Interestingly, in southern Africa, fresh leaves of *M. longifolia* are used as an analgesic to relieve labour pains, stomach pains and headaches (Hutchings et al. 1996; Mhlongo 2019). The plant is also used as a general medicine and the leaves are prepared in water as herbal tea (Van Wyk and Gericke 2018). Therefore, the findings from our study may support the traditional medicinal use of *M. longifolia* as an analgesic by regulating the synthesis of pain-producing molecules through substantial inhibition of IL-1β levels. The leaf methanol extract of *M. longifolia,* together with its major constituent eucalyptol, have protective effects against acetic acid-induced colitis in rats by significantly reducing the up-relation of serum IL-6 and TNF-α levels (Murad et al. 2016). The leaf methanol extract and essential oils of *M. longifolia* exhibited potent anti-inflammatory effects by NO scavenging and reducing inducible nitric oxide synthase and tumour necrosis factor-α mRNA expression in LPS-stimulated macrophages (J774) (Karimian et al. 2013). Although the chemical composition of *M. longifolia* essential oils has been well studied, relatively few studies have focused on their inflammatory pain-related properties (Farzaei et al. 2017; Eftekhari et al. 2021).

The different solvent extracts used in this study, greatly influenced the therapeutic effects of *Plumbago auriculata, W. salutaris* and *Z. albomaculata,* the aqueous extracts of the medicinal plants exhibited immunostimulant effects, while the ethanol extracts displayed substantial cytokine inhibition activity in LPS-induced RAW 264.7 macrophage cells. The aqueous extract of *P. auriculata* enhanced the release of IL-6 by up to 418.2% compared to the control, whilst the ethanol extract showed a significant decrease to 18.2% compared to the control (Fig. 3). The anti-inflammatory properties of the bioactive constituents of *P. auriculata* have recently been reported (Melk et al. 2021; Sherif et al. 2022; Selim et al. 2022). The roots contain the naphthoquinones epi-isoshinanolone and plumbagin, the steroids sitosterol and 3-O-glucosylsitosterol, as well as plumbagic and palmitic acids (De Paiva et al. 2005). High performance liquid chromatography (HPLC) analysis of ethanol extracts of aerial parts of this plant revealed the presence of sixteen compounds, with gallic acid, chlorogenic acid and catechin identified as major constituents (Melk et al. 2021). Reported anti-inflammatory properties of gallic acid include inhibition of LPS-induced nitric oxide (NO), prostaglandin E-2 (PGE-2) and IL-6 production in RAW264.7 cells (BenSaad et al. 2017). Thus, the IL-6 modulatory activity of *P. auriculata* observed in our study may be influenced by these chemical constituents, although this remains to be verified.

The leaves of *P. auriculata* are used traditionally to repair broken bones and taken as a snuff for headaches (Hutchings et al. 1996; Van Wyk et al. 2009). Interleukin-6 plays a key role in promoting osteomyelitis through signalling receptor protein gp130 binding (Scheller et al. 2011). Mouse models of arthritis, including antigen-induced arthritis and collagen-induced arthritis, showed IL-6 deficient mice to be completely protected against rheumatoid arthritis (Scheller et al. 2011). The immunomodulatory properties of *P. auriculata* leaves were also evaluated by other authors in in vivo models. The ethanol extracts of aerial parts exhibited potent hepatoprotective effects by significantly suppressing TNF-α and IL-6 levels in thioacetamide (TAA)-induced liver fibrosis in rats, thus restoring liver function (Selim et al. 2022). Additionally, the methanol extract of the aerial parts of *P. auriculata* and its bioactive constituents exhibited potent anti-inflammatory activity evaluated in carrageenan-induced paw oedema model in rats (Sherif et al. 2022). The authors isolated and identified 16 compounds of which β-amyrin, β-sitosterol, β-sitosterol-3-O-b-d-glucoside, and the glycosylated flavonoids and biflorin showed the most significant analgesic effects through inhibition of the paw swelling.

Interferons are inflammatory cytokines that regulate various transcription factors through STATs signalling pathway (Rauch et al. 2013). Increased serum levels of IFN-γ were reported in patients suffering from systemic lupus erythematous, inflammatory bowel diseases, multiple sclerosis, and rheumatoid arthritis (Ito et al. 2006; Rauch et al. 2013; Majoros et al. 2017). Furthermore, histological study of mice intestine following oral administration of dextran sodium sulphate damaged mucosal epithelium and showed increased infiltration of inflammatory cells (Ito et al. 2006). The leaf extracts of the eight medicinal plants tested in this study displayed strong inhibition of IFN-γ levels in LPS-stimulated RAW 264.7 cells compared to the control, particularly the ethanol extracts. In contrast, most of the aqueous extracts favoured substantial up-regulation of IFN-γ levels to the control, and therefore have pro-inflammatory effects.

The extracts of *T. sericea* and *T. riparia* significantly decreased pro-inflammatory cytokines levels in LPS-induced RAW 264.7 cells, with limited toxicities in the MTS assay. The reported biological properties of *T. sericea* include anti-HIV, antibacterial, anti-fungal, wound healing, anticancer, lipolytic, antiparasitic, anti-inflammatory and antioxidant activities (Mongalo et al. 2016). The leaves are mainly used traditionally against gastrointestinal and respiratory tract disorders (Hutchings et al. 1996; Van Wyk et al. 2009). However, unspecified parts of the plant are also used to treat leg pains in Namibia (Cheikhyoussef et al. 2011). The ethanol leaf extract of *T. sericea* showed potent anti-inflammatory properties through inhibition of the pro-inflammatory mediators IL-1β, IL-6 and TNF-α. Furthermore, no toxicities were observed in the MTS assay at 1.25 mg/mL against RAW 264.7 cells. Very little is known about the toxicity and inflammatory enzyme inhibition activity of *T. sericea* (Mongalo et al. 2016). This is the first report of cytokine inhibition assays for this species. There is a need to further understand the mode of action and in vivo cytotoxicity properties of this plant.

*Tetradenia riparia* is an important Zulu medicinal plant that is widely used for treatment of several disorders. Leaf infusions are used to treat swellings, backache, and chest pains, whilst powdered leaves are directly inhaled as a snuff for headaches (Van Wyk et al. 2009; Mhlongo 2019). The ethanol extract of *T. riparia* has broad-spectrum cytokine inhibition activity, with the greatest inhibition against IL-6 and TNF-α secretion. Notably, TNF-α regulates many critical processes of tumour promotion and progression, with clinically elevated serum levels noted in individuals suffering from pre-neoplastic and malignant diseases (Landskron et al. 2014). One study reported that the leaf essential oils of *T. riparia* induced IL-1β and IL-6 secretion without altering TNF-α mRNA expression in LPS-stimulated murine macrophage cells following a 3 h incubation (Demarchi et al. 2016). Furthermore, cytotoxicity evaluation of the essential oils at 30 ng/mL showed 90% cell viability in the XTT (sodium 3′-[1-(phenylaminocarbonyl)-3,4-tetrazolium]-bis (4-methoxy 6-nitro) benzene sulfonic acid hydrate) assay (Demarchi et al. 2016). Antiproliferative effects of *T. riparia* leaf extracts were reported against various cancer cell lines (DU145)-prostate cancer cells (DU145), HCC-breast cancer cells (HCC) and Hela-cervical cancer cells (Hela) without showing toxicities in normal cells (Vero monkey kidney cell line) in the MTT assay (Shimira 2022). No further studies on the pro-inflammatory cytokine inhibition assay and in vivo anti-inflammatory properties of the plant were found.

The aqueous extracts of several species screened herein displayed substantial suppression of pro-inflammatory cytokines and chemokines. Of particular note are the aqueous extracts of *H. procumbens*, *M. comosus* and *T. riparia,* which significantly inhibited the secretion of pro-inflammatory cytokine levels in LPS-induced RAW 264.7 cells by up to 80% inhibition compared to the control. Decoctions and infusions of *H. procumbens* are traditionally taken orally as a daily tonic and used to treat various auto-immune conditions (Mncwangi et al. 2012; Van Wyk and Gericke 2018). *Harpagophytum procumbens* is popular southern African medicinal plant used traditionally for chronic inflammation, rheumatoid arthritis, and as analgesics (Stewart and Cole 2005; Mncwangi et al. 2012). Isolated compounds and plant parts are commercialised globally for the degenerative rheumatoid arthritis, osteoarthritis, tendonitis, kidney inflammation and cardiovascular diseases (Stewart and Cole 2005; Mncwangi et al. 2012; Menghini et al. 2019; Gxaba and Manganyi 2022). Inflammatory cytokine studies in LPS-stimulated cells, the mechanisms of action, as well as in vivo analgesic and anti-inflammatory properties of *H. procumbens* have been relatively well studied (Inaba et al. 2010; Mncwangi et al. 2012; Menghini et al. 2019; Gxaba and Manganyi 2022). In addition, multiple pre-clinical and clinical safety studies have been documented in a recent (reviewed by Menghini et al. 2019). The aqueous extracts of *H. procumbens* were safe and effective against arthritis following a 3-month trial conducted in 75 patients with hip or knee arthritis (Wegener and Lüpke 2003). Another study performed on 259 patients who orally administered dried plant extracts of *H. procumbens* (960 mg/day) for 8 weeks reported substantially reduced stiffness with improvements in joint pain (Menghini et al. 2019).

The potent anti-inflammatory properties of *M. comosus* against all the tested cytokines and chemokines in this study, strongly supports the traditional use against chronic inflammatory disorders. However, concerns about the toxic properties of the plant highlights the need for extensive acute and chronic toxicity studies. The MTS assay results of our study indicate potential toxicity of *M. comosus* ethanol extract at 1.25 mg/mL against human dermal fibroblast with < 50% cell viability (*p* < 0.005), whilst the aqueous extract was non-toxic against both HDF and RAW 264.7 cells. Notably, there are relatively few studies reporting the cytotoxic properties of *M. comosus* (Maroyi 2019). Acetone extracts of the leaves (concentration not specified) was non-toxic in the brine shrimp assay (McGaw et al. 2005). In contrast, an ethanol extract of the leaves displayed toxicity against Vero cells, with LC_50_ of 51.4 μg/mL in XTT toxicity assays (Heyman et al. 2009). The toxic properties of the plant have been ascribed to cardiac glycosides that have been identified as bufadienolides (Van Wyk et al. 2009; Maroyi 2019). Bufadienolides (including 16β-formyloxymelianthugenin, 2β-acetoxymelianthusigenin, 2β-hydroxy-3β,5β-di-O-acetylhellebrigenin, and 2β-acetoxy-5β-*O*-acetylhellebrigenin) isolated from the leaves exhibited potent toxicities against multiple cancer cells in resazurin reduction assays (Bedane et al. 2020).

Although the toxic properties of *M. comosus* have been reported mostly for non-polar constituents, it is important to note that the aqueous extract exhibited significant inhibition against several pro-inflammatory cytokines and chemokines in LPS-stimulated RAW 264.7 cells, particularly for IL-6 levels compared to the control. *Melianthus comosus* is an important Khoi medicinal plant prepared as an infusion of fresh leaves in water and taken externally as a wash to treat backache and rheumatic joints (Van Wyk et al. 2009). The leaves, leaf juice and roots are also used to treat cancer, stroke, septic wounds, abscesses and boils, inflammation, swellings, painful feet, headaches, and toothache (Maroyi 2019). Our findings support the traditional method of *M. comosus* prepared in water and taken as infusions and decoctions to treat inflammatory pain-related disorders. Potential drug leads targeting IL-6, the IL-6 receptor, or Janus kinase (JAK)–signal transducer have been FDA approved for the treatment of inflammatory conditions or myeloproliferative neoplasms and are being further evaluated in patients with haematopoietic malignancies and in those with solid tumours (Johnson et al. 2018). The significant inhibition of *M. comosus* extracts against IL-1β, TNF-α and IL-6 makes it a potential therapeutic target against multiple immune disorders. Although some anti-inflammatory properties of the plant have been previously reported against COX and LOX inhibition (Frum and Viljoen 2006; Adebayo et al. 2015; Maroyi 2019; Khumalo et al. 2022), this is the first report of cytokine and chemokine inhibition activity of this plant.

The findings from our study showed significant inhibition of MCP-1 and MIP-2 secretion in LPS-stimulated RAW 264.7 macrophages. Upon tissue damage, chemokines function in the recruitment of leukocytes from the blood vessels via G-protein-coupled receptor, signalling and facilitating migration to the inflamed area (Deshmane et al. 2009). MCP-1is among the most studied chemokines and is a therapeutic target in the treatment of various diseases, including multiple sclerosis, rheumatoid arthritis, atherosclerosis, allergic conditions, and insulin-resistant diabetes (Deshmane et al. 2009; Yadav et al. 2010). Animal models (genetically deficient mice, antibody- or inhibitor-mediated neutralization in mice) that neutralised overexpression of MCP-1 in these disease states showed substantially improved recovery rates (Deshmane et al. 2009; Yadav et al. 2010).

## Conclusions

This study evaluated the anti-inflammatory properties of leaf extracts prepared from eight selected medicinal plants of southern African that are used traditionally to treat pain and inflammation. The medicinal plant extracts exhibited potential anti-inflammatory effects through substantial inhibition of pro-inflammatory cytokines and chemokine secretion. However, opposite effects towards the inflammatory response were noted for different extracts of the same species. The aqueous extracts of *P. auriculata*, *W. salutaris* and *Z. albomaculata* displayed pro-inflammatory effects by significantly up-regulating cytokine levels beyond that of the control, whilst the ethanol extracts showed significant inhibition compared to the control. Extensive work is required to investigate the chemical constituents of the aqueous and ethanol extracts to identify compounds that may have immunomodulatory effects. It is also important to note that whilst most of the species are widely used traditionally against inflammatory disorders, very few studies have explored the possible mechanisms of action and substantial further work is needed.

## Data Availability

Enquiries about data availability should be directed to the authors.

## References

[CR1] Adebayo SA, Dzoyem JP, Shai LJ, Eloff JN (2015). The anti-inflammatory and antioxidant activity of 25 plant species used traditionally to treat pain in southern Africa. BMC Complement Altern Med.

[CR2] Bedane KG, Brieger L, Strohmann C, Seo EJ, Efferth T, Spiteller M (2020). Cytotoxic bufadienolides from the leaves of a medicinal plant *Melianthus comosus* collected in South Africa. Bioorg Chem.

[CR3] BenSaad LA, Kim KH, Quah CC, Kim WR, Shahimi M (2017). Anti-inflammatory potential of ellagic acid, gallic acid and punicalagin A&B isolated from *Punica granatum*. BMC Complement Altern Med.

[CR4] Chao CH, Wen ZH, Wu YC, Yeh HC, Sheu JH (2008). Cytotoxic and anti-inflammatory cembranoids from the soft coral *Lobophytum crassum*. J Nat Prod.

[CR5] Cheikhyoussef A, Shapi M, Matengu K, Ashekele HM (2011). Ethnobotanical study of indigenous knowledge on medicinal plant use by traditional healers in Oshikoto region, Namibia. J Ethnobiol Ethnomed.

[CR6] De Paiva SR, Figueiredo MR, Kaplan MAC (2005). Isolation of secondary metabolites from roots of *Plumbago auriculata* Lam. by counter current chromatography. Phytochem Anal.

[CR7] Demarchi IG, Terron MDS, Thomazella MV, Mota CA, Gazim ZC, Cortez DAG, Aristides SMA, Silveira TGV, Lonardoni MVC (2016). Antileishmanial and immunomodulatory effects of the essential oil from *Tetradenia riparia* (Hochstetter) Codd. Parasite Immunol.

[CR8] Deshmane SL, Kremlev S, Amini S, Sawaya BE (2009). Monocyte chemoattractant protein-1 (MCP-1): an overview. J Interferon Cytokine Res.

[CR9] Dinarello CA (2011). Interleukin-1 in the pathogenesis and treatment of inflammatory diseases. Blood.

[CR10] Dinarello CA (2018). Overview of the IL-1 family in innate inflammation and acquired immunity. Immunol Rev.

[CR11] Doğanyiğit Z, Üner AK (2021). Immature neuronal markers: NeuroD1, doublecortin, PSA-NCAM and their use to immunochemistry. Res Rev Health Sci..

[CR12] Eftekhari A, Khusro A, Ahmadian E, Dizaj SM, Hasanzadeh A, Cucchiarini M (2021). Phytochemical and nutra-pharmaceutical attributes of *Mentha* spp.: a comprehensive review. Arab J Chem.

[CR13] Elgorashi EE, McGaw LJ (2019). African plants with in vitro anti-inflammatory activities: a review. S Afr J Bot.

[CR14] Farzaei MH, Bahramsoltani R, Ghobadi A, Farzaei F, Najafi F (2017). Pharmacological activity of *Mentha longifolia* and its phytoconstituents. J Tradit Chin Med.

[CR15] Fitzgerald AA, LeClercq SA, Yan A, Homik JE, Dinarello CA (2005). Rapid responses to anakinra in patients with refractory adult-onset Still's disease. Arthritis Rheumatol.

[CR16] Frum Y, Viljoen AM (2006). *In vitro* 5-lipoxygenase and antioxidant activities of South African medicinal plants commonly used topically for skin diseases. Skin Pharmacol Physiol.

[CR17] Fullerton JN, Gilroy DW (2016). Resolution of inflammation: a new therapeutic frontier. Nat Rev Drug Discov.

[CR18] Fürst R, Zündorf I (2014). Plant-derived anti-inflammatory compounds: hopes and disappointments regarding the translation of preclinical knowledge into clinical progress. Mediators Inflamm.

[CR19] Gxaba N, Manganyi MC (2022). The fight against infection and pain: devil’s claw (*Harpagophytum procumbens*) a rich source of anti-inflammatory activity: 2011–2022. Molecules.

[CR20] Heyman HM, Hussein AA, Meyer JJM, Lall N (2009). Antibacterial activity of South African medicinal plants against methicillin resistant *Staphylococcus aureus*. Pharm Biol.

[CR21] Hirano T (2021). IL-6 in inflammation, autoimmunity and cancer. Int Immunol.

[CR22] Hutchings A, Scott AH, Lewis G, Cunningham A (1996). Zulu medicinal plants: an inventory.

[CR23] Inaba K, Murata K, Naruto S, Matsuda H (2010). Inhibitory effects of devil's claw (secondary root of *Harpagophytum procumbens*) extract and harpagoside on cytokine production in mouse macrophages. J Nat Med.

[CR24] Ito R, Shin-Ya M, Kishida T, Urano A, Takada R, Sakagami J, Imanishi J, Kita M, Ueda Y, Iwakura Y, Kataoka K (2006). Interferon-gamma is causatively involved in experimental inflammatory bowel disease in mice. Clin Exp Immunol.

[CR25] Ji RR, Nackley A, Huh Y, Terrando N, Maixner W (2018). Neuroinflammation and central sensitization in chronic and widespread pain. Anesthesiology.

[CR27] Johnson DE, O'Keefe RA, Grandis JR (2018). Targeting the IL-6/JAK/STAT3 signalling axis in cancer. Nat Rev Clin Oncol.

[CR28] Kalotra S, Kaur G (2021). PSA mimetic 5-nonyloxytryptamine protects cerebellar neurons against glutamate induced excitotoxicity: an in vitro perspective. Neurotoxicology.

[CR29] Kalotra S, Saini V, Singh H, Sharma A, Kaur G (2020). 5-Nonyloxytryptamine oxalate–embedded collagen–laminin scaffolds augment functional recovery after spinal cord injury in mice. Ann N Y Acad Sci.

[CR30] Karimian P, Kavoosi G, Amirghofran Z (2013). Anti-inflammatory effect of *Mentha longifolia* in lipopolysaccharide-stimulated macrophages: reduction of nitric oxide production through inhibition of inducible nitric oxide synthase. J Immunotoxicol.

[CR31] Khumalo GP (2018) An inventory of the most popular medicinal barks sold on Johannesburg muthi markets and the antimicrobial activity of selected extracts and isolated chemical compounds. MSc thesis: University of Johannesburg

[CR32] Khumalo GP, Van Wyk B-E, Feng Y, Cock IE (2022). A review of the traditional use of Southern African medicinal plants for the treatment of inflammation and inflammatory pain. J Ethnopharmacol.

[CR34] Laczko R, Chang A, Watanabe L, Petelo M, Kahaleua K, Bingham JP, Csiszar K (2020). Anti-inflammatory activities of *Waltheria indica* extracts by modulating expression of IL-1B, TNF-α, TNFRII and NF-κB in human macrophages. Inflammopharmacology.

[CR35] Lai KM, Wang JH, Lin SC, Wen Y, Wu CL, Su JH, Chen CC, Lin CC (2022). Crassolide induces G2/M cell cycle arrest, apoptosis, and autophagy in human lung cancer cells via ROS-mediated ER stress pathways. Int J Mol Sci.

[CR36] Landskron G, De la Fuente M, Thuwajit P, Thuwajit C, Hermoso MA (2014). Chronic inflammation and cytokines in the tumour microenvironment. J Immunol Res.

[CR37] Lin CC, Chang YK, Lin SC, Su JH, Chao YH, Tang KT (2021). Crassolide suppresses dendritic cell maturation and attenuates experimental antiphospholipid syndrome. Molecules.

[CR38] Liu JK (2014). The history of monoclonal antibody development—progress, remaining challenges and future innovations. Ann Med Surg.

[CR39] Magwede K, Van Wyk B-E, Van Wyk AE (2019). An inventory of vhaVenḓa useful plants. S Afr J Bot.

[CR40] Majoros A, Platanitis E, Kernbauer-Hölzl E, Rosebrock F, Müller M, Decker T (2017). Canonical and non-canonical aspects of JAK–STAT signaling: lessons from interferons for cytokine responses. Front Immunol.

[CR42] Mander M, Ntuli L, Diederichs N, Mavundla K (2007). Economics of the traditional medicine trade in South Africa: health care delivery. S Afr Health Rev.

[CR43] Maroyi A (2019). A review of the ethnomedicinal uses, phytochemistry and pharmacological properties of *Melianthus comosus* Vahl. Int J Pharm Sci Res.

[CR44] McGaw LJ, Eloff JN, Meyer JJM (2005). Screening of 16 poisonous plants for antibacterial, anthelmintic and cytotoxic activity *in vitro*. S Afr J Bot.

[CR45] Melk MM, El-Hawary SS, Melek FR, Saleh DO, Ali OM, El Raey MA, Selim NM (2021). Nano zinc oxide green-synthesized from *Plumbago auriculata* lam. alcoholic extract. Plants.

[CR46] Menghini L, Recinella L, Leone S, Chiavaroli A, Cicala C, Brunetti L, Vladimir-Knežević S, Orlando G, Ferrante C (2019). Devil's claw (*Harpagophytum procumbens*) and chronic inflammatory diseases: a concise overview on preclinical and clinical data. Phytother Res.

[CR47] Mhlongo LS (2019) The medicinal ethnobotany of the Amandawe area in KwaCele, KwaZulu-Natal, South Africa. MSc Thesis. University of Johannesburg.

[CR48] Mncwangi N, Chen W, Vermaak I, Viljoen AM, Gericke N (2012). Devil's Claw—a review of the ethnobotany, phytochemistry and biological activity of *Harpagophytum procumbens*. J Ethnopharmacol.

[CR49] Mongalo NI, McGaw LJ, Segapelo TV, Finnie JF, Van Staden J (2016). Ethnobotany, phytochemistry, toxicology and pharmacological properties of *Terminalia sericea* Burch. ex DC. (Combretaceae)—a review. J Ethnopharmacol.

[CR50] Murad HA, Abdallah HM, Ali SS (2016). *Mentha longifolia* protects against acetic-acid induced colitis in rats. J Ethnopharmacol.

[CR51] Ndhlala AR, Stafford GI, Finnie JF, Van Staden J (2011). Commercial herbal preparations in KwaZulu-Natal, South Africa: the urban face of traditional medicine. S Afr J Bot.

[CR52] Pier GB (2007). *Pseudomonas aeruginosa* lipopolysaccharide: a major virulence factor, initiator of inflammation and target for effective immunity. Int J Med Microbiol.

[CR53] Pohanka A, Broberg A, Johansson M, Kenne L, Levenfors J (2005). Pseudotrienic acids A and B, two bioactive metabolites from *Pseudomonas* sp. MF381-IODS. J Nat Prod.

[CR54] Qin S, Xiao W, Zhou C, Pu Q, Deng X, Lan L, Liang H, Song X, Wu M (2022). *Pseudomonas aeruginosa*: pathogenesis, virulence factors, antibiotic resistance, interaction with host, technology advances and emerging therapeutics. Signal Transduct Target Ther.

[CR56] Rauch I, Müller M, Decker T (2013). The regulation of inflammation by interferons and their STATs. Jak-Stat.

[CR57] Saini V, Lutz D, Kataria H, Kaur G, Schachner M, Loers G (2016). The polysialic acid mimetics 5-nonyloxytryptamine and vinorelbine facilitate nervous system repair. Sci Rep.

[CR58] Scheller J, Chalaris A, Schmidt-Arras D, Rose-John S (2011). The pro-and anti-inflammatory properties of the cytokine interleukin-6. Biochim Biophys Acta-Mol Cell Res.

[CR59] Selim NM, Melk MM, Melek FR, Saleh DO, Sobeh M, El-Hawary SS (2022). Phytochemical profiling and anti-fibrotic activities of *Plumbago indica* L. and *Plumbago auriculata* Lam. in thioacetamide-induced liver fibrosis in rats. Sci Rep.

[CR60] Sherif AE, Amen Y, Shimizu K (2022). Validation of the potential anti-inflammatory activity of *Plumbago auriculata* Lam. S Afr J Bot.

[CR61] Shimira F (2022). *Tetradenia riparia*, an ethnobotanical plant with diverse applications, from antimicrobial to anti-proliferative activity against cancerous cell lines: a systematic review. J Herb Med.

[CR62] Stewart KM, Cole D (2005). The commercial harvest of devil's claw (*Harpagophytum* spp.) in southern Africa: the devil's in the details. J Ethnopharmacol.

[CR63] Van Der Hooft JJ, Goldstone RJ, Harris S, Burgess KE, Smith DG (2019). Substantial extracellular metabolic differences found between phylogenetically closely related probiotic and pathogenic strains of *Escherichia coli*. Front Microbiol.

[CR64] Van Wyk B-E, Gericke N (2018). People’s plants: a guide to useful plants of southern Africa.

[CR66] Van Wyk B-E, Oudtshoorn B, Gericke N (2009). Medicinal plants of South Africa.

[CR67] Wegener T, Lüpke NP (2003). Treatment of patients with arthrosis of hip or knee with an aqueous extract of devil's claw (*Harpagophytum procumbens* DC.). Phytother Res.

[CR68] Williams VL, Lawes MJ, Eeley HAC, Shackleton CM (2004). Trade and socio-economic value of forest and woodland reserves within the medicinal plan market in Johannesburg. Indigenous forests and woodlands in South Africa: policy, people and practice.

[CR69] Yadav A, Saini V, Arora S (2010). MCP-1: chemoattractant with a role beyond immunity: a review. Clin Chim Acta.

